# Particle movements provoke avalanche-like compaction in soft colloid filter cakes

**DOI:** 10.1038/s41598-021-92119-w

**Published:** 2021-06-18

**Authors:** Arne Lüken, Lucas Stüwe, Johannes Lohaus, John Linkhorst, Matthias Wessling

**Affiliations:** 1 Chemical Process Engineering AVT.CVT, RWTH Aachen University, Forckenbeckstr. 51, 52074 Aachen, Germany; 2 DWI-Leibniz Institute for Interactive Materials, Forckenbeckstr. 50, 52074 Aachen, Germany

**Keywords:** Chemical engineering, Biomedical materials, Mechanical properties

## Abstract

During soft matter filtration, colloids accumulate in a compressible porous cake layer on top of the membrane surface. The void size between the colloids predominantly defines the cake-specific permeation resistance and the corresponding filtration efficiency. While higher fluxes are beneficial for the process efficiency, they compress the cake and increase permeation resistance. However, it is not fully understood how soft particles behave during cake formation and how their compression influences the overall cake properties. This study visualizes the formation and compression process of soft filter cakes in microfluidic model systems. During cake formation, we analyze single-particle movements inside the filter cake voids and how they interact with the whole filter cake morphology. During cake compression, we visualize reversible and irreversible compression and distinguish the two phenomena. Finally, we confirm the compression phenomena by modeling the soft particle filter cake using a CFD-DEM approach. The results underline the importance of considering the compression history when describing the filter cake morphology and its related properties. Thus, this study links single colloid movements and filter cake compression to the overall cake behavior and narrows the gap between single colloid events and the filtration process.

## Introduction

Membrane filtration is a unit operation in chemical processes to efficiently separate colloids from aqueous suspensions applied in, e.g., water treatment, food processing, or bio processing. During filtration, the colloids accumulate on the feed side surface of the membrane in a cake layer. This cake layer generates an additional resistance on top of the membrane’s resistance and reduces the performance of the overall filtration process^1^. To optimize filtration performance and predict cake properties, intrinsic knowledge of the phenomena occurring inside the cake on a colloid-scale is essential.

During the filtration of biological suspensions, such as applied in food processing^2^ or membrane bioreactors^3^, the filtered matter is soft and deformable. In contrast to hard particle filtration, the soft particle cake layer is compressible, such that the specific cake resistance depends on the filtration time and the applied flux^4^. Additionally, the soft cake layer is not packed uniformly but shows an increasing packing density towards the membrane^5^. Accordingly, the specific cake resistance increases over the filter cake thickness and reaches its maximum at the most compressed gel layer next to the membrane^6^. In soft matter microfiltration applications, this gel layer often dominates the pressure loss of the overall process^7–9^.

For explaining gel layer formation, we need to consider the general phenomena during compression of a collection of soft colloids. This compression strongly depends on the local colloid concentration and can be described in three stages. In the first stage, concentration is low, and single colloids are separated from each other. The colloids behave like hard spheres rearranging their position and filling up large voids in the cake^10^. In the second glass transition stage, the concentration is higher. The colloids are in a fixed position inside the collection and start deforming and narrowing the neighboring colloids’ voids. Many different physical phenomena ranging from thermal agitation to compression and deformation^9,11^, as well as interpenetration and crystallization^12,13^ depend on the particle’s softness and charge and influence the cake’s morphology and the corresponding cake resistance^14,15^. One phenomenon of this glass transition stage is that the packing’s morphology and coordination depend on the previous filtration time and applied force^16,17^. The particles stick in an amorphous geometrical frustration, which can reach a higher packing density by mechanical agitation induced rearrangement^18^. The particle interactions, as well as their shape and size, significantly affect the rearrangement kinetics and the resulting packing^19^. In the last stage, at very high compression, the colloids form a homogeneous material without voids. The packing still behaves compressible by water expulsion like a polymer gel^20^.

Stage one and stage two networks occur in a filtration process during cake formation, where fluid permeates the compressed filter cake and the voids between the colloids. The void size mainly influences the cake-specific fluid resistance, and the pressure loss correlates to the inverse void-radius to the power of four (Hagen–Poiseuille). Subsequently, minor void size changes induced by particle deformation and cake compression affect the permeation characteristic tremendously^11,21,22^. The most compressed gel layer with the smallest voids next to the membrane dominates the overall pressure loss^5^. This dense gel layer might become a stage three network without voids when filtering tiny and soft colloids at high compression or when particles break by compression. In this case, the fluid needs to permeate through the compressed colloid material, which has higher fluid resistances than the filter cake’s porous structure. Thus, the gel layer permeation properties dominate the overall cake resistance^23^.

The soft matter multi-stage cake behavior and the subsequent filtration process is often inaccurately described by models, such as the conventional cake filtration theory^7,14^. There exist a variety of empirical and semi-empirical models, which macroscopically describe softness and compression effects of the filter cake on the permeation^7,24–26^. These models are very valuable to describe and to design filtration processes. The interplay between the reversible/irreversible compression and hydrodynamic permeation is determined by the micromechanics of the individual soft particles, which is difficult to analyze with these macroscopic models. Therefore, the evaluation of the micro-mechanics of the particles inside the filter cake is of great interest. Simulation tools combining particle mechanics coupled with fluid dynamics help to decode the complex reversible and irreversible compression phenomena inside the filter cake of soft particles. These methods potentially enable to connect the individual soft particles’ properties and the empirical parameters which still remains unsolved.

Microfluidic filtration of colloids at porous structures have proven to be a powerful tool for mimicking real filtration processes and visualizing cake morphology on particle scale^27^. Hard spherical colloids were flushed through tight microfluidic pore-mimicking constrictions inside poly-(dimethylsiloxane) (PDMS) channels, and the phenomena of particle adhesion on the pore surface and the consequent pore-blocking were studied using visual methods^28^. The pore geometry, as well as the foulant and the fluid-properties, were studied. The studies reveal the importance and the influence of particle-particle and particle-wall interactions on particle adsorption and pore-blocking^29–31^. In contrast to hard particles, soft particles can adapt to the pore geometries by deformation and, therefore, show a smaller pore-blocking tendency^32,33^. The microfluidic observation method was applied for pore phenomena and for studying the filter cake in a fouling layer. Different studies investigate the influence of the cake morphology on permeation^34,35^, the deformation of single microgels during cake compression^11^, and the relaxation and removal of filter cakes by backflushing^29,36,37^. These microfluidic studies display the complexity of the phenomena influencing single particles’ behavior during micro-and ultrafiltration by studying single events during pore blocking, cake formation, or cake compression. However, transferring single colloid phenomena to real membrane processes lacks the description of how single colloid events, such as colloid deformation, affect the overall cake morphology and its properties.

In this work, we study the influence of single-particle movements on the cake properties in microfluidic soft filter cakes. Hence, we filter 27 ${\upmu }$m sized soft polyethylene-glycol (PEG) microgels in front of porous microfluidic structures. We study the impact of single-particle movements inside the filter cake during filtration by image analysis. Thus, we differentiate particle movements that induce avalanche-like v-shaped displacement of a particle network inside the cake and minor movements of single particles not affecting the cake morphology. After cake buildup, we compress the cake by applying pressure stepping experiments, differentiate reversible and irreversible cake compression, and localize the spatial position of the two effects. Finally, we explain and confirm the findings of irreversible compression using CFD-DEM simulations.

## Results and discussion

**Figure 1:**
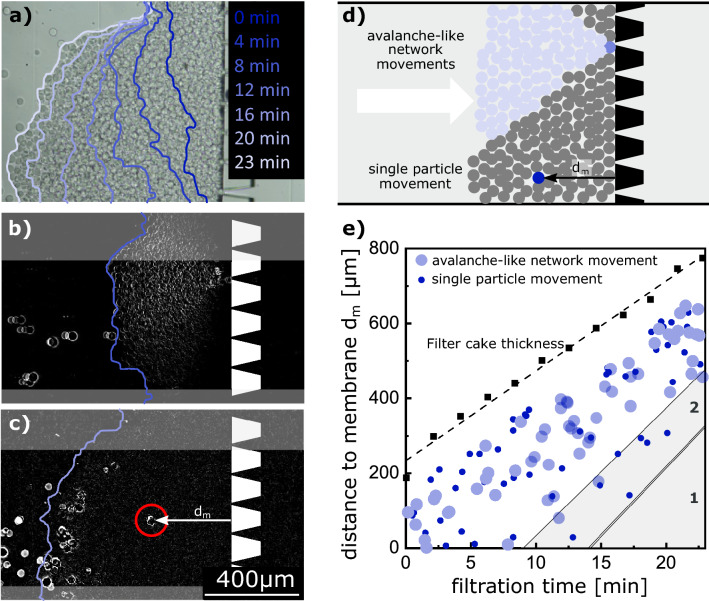
Evolution of a filter cake of 27 ${\upmu }$m sized PEG microgels inside a microfluidic PDMS channel during constant pressure filtration. The lines in (**a**) indicate the cake surface at the respective time. Visualization of the local cake movements by microgel rearrangement with avalanche-like network movements (**b**), and single-particle movements (**c**). We overlaid the filter cake surface at that respective time as a blue line and the porous filtration structure in white for better orientation. Illustration of the two types of rearrangement occurring in the filter cake (**d**). Position of the rearrangements (blue dots) in the filter cake at the respective filtration time and the filter cake thickness (black squares) (**e**).

Soft PEG microgels with a diameter of approx. 27 ${\upmu }$m are fabricated by microfluidic emulsion polymerization and are filtrated inside a microfluidic PDMS filtration channel using a porous filter structure. The filter structure in the channel consists of several filtration blocks with a trapezoidal cross-section as indicated in Fig. 1b–d. The gaps between the filtration blocks are 3 ${\upmu }$m such that the microgels are entirely rejected and do not permeate by deformation. The channel’s height is 150 ${\upmu }$m, such that more than eight microgel layers fit in the filter cake on top of each other. A detailed geometrical description can be found in the method section and as a 3D stl-model in the supplementary information (SI1_ChannelGeometry.stl).

### Cake mobility during buildup

We studied the mobility of microgels in a filter cake during filter cake formation at constant pressure. Therefore, we filtrated microgels for about 23 min and visualized the cake growth in Fig. 1a. Inside the cake, the microgels change their initial position to fill the voids in the arrangement. We applied a time filter to the cake formation video to improve the visualization of particle movement in the filter cake. The time filter subtracts the average of the previous three frames from each frame. The results is a mostly dark video. Areas without brightness change over the previous three images remain dark, whereas areas in which the pixel information change over the previous three images appear bright. Hence, bright areas highlight particle movements in the bulk and in the filter cake. Moving particles in the bulk change their position over the previous frames and appear as trajectories. One-time movements in the filter cake appear as bright spots. Figure 1b,c showcase two selected frames of the edited video revealing two different phenomenological types of morphology change. The first type of change describes an avalanche-like displacement of particle networks over the whole filter cake. This type is characterized by a v-shaped geometry ranging from a single microgel on the right side to the cake surface on the left side of the image. These network movements are induced by a single microgel movement on the right side in the filter cake, which causes the following layers to rearrange (see Fig. 1b). The second type of morphology change is the single-particle movement, where only one microgel modifies its position in the particle arrangement. In contrast to the first type, no avalanche-like rearrangement follows, and neighboring microgels remain at their initial position (see Fig. 1c). Both types of morphology change are illustrated in Fig. 1d.

In Fig. 1e we extracted the particle movements of the cake build-up video, analyzed them in terms of their distance to the membrane, and differentiated the above-mentioned avalanche-like movements from single-particle movements. The dashed line shows the continuous filter cake growth over time. We excluded wall effects and non-uniform cake thicknesses by omitting the particle movements in the upper 300 and lower 100 pixels of the images, such as indicated in the grayed areas in Fig. 1c,d. Movements close to the cake surface during the initial attachment of incoming microgels also appear bright (compare to Fig. 1c next to the cake boundary line). As those microgels have fewer neighbors and higher freedom of movement, rearrangement in this region is observed with each inflowing microgel. However, these movements do not play a decisive role in the filter cake’s morphology and are therefore also omitted. Hence, Fig. 1e shows a white gap between the filter cake surface and the defect positions.

At the beginning of the experiment, during the first 10 min, the system represents a homogeneous state. Rearrangements are initiated by flow-induced mechanical agitation, such that single-particle and avalanche-like network movements are both occurring and evenly spread over the whole filter cake. Several events cluster simultaneously in a short time, suggesting that rearranged microgels open novel voids subsequently filled by the following microgels. With ongoing filtration time, the defect positions shift towards the cake surface, as seen in the grayed region 1 of Fig. 1e. This shift indicates that the total number of rearrangements in a layer is limited. Each rearrangement increases packing density towards the membrane, reducing remaining voids and further rearrangements become unlikely. Next to region 1, we see a decreased probability of avalanche-like network movements in region 2. In this region 2, the packing density is already on a high level, and load-bearing microgels have found their stable position, such that only local single-particle movement of non-loaded microgels occurs.

### Cake mobility during compression and relaxation

**Figure 2:**
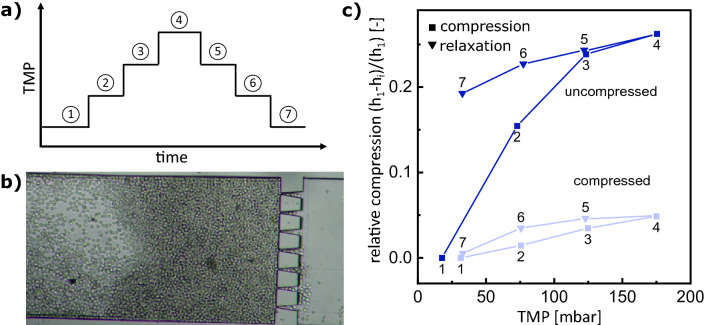
Cake thickness during pressure stepping experiment. Experimental procedure of the applied transmembrane pressure (TMP) steps (**a**). Light microscopy image of the microfluidic membrane channel and the filter cake during pressure step 1 (**b**). Cake compression relative to the initial thickness $\frac{h_1-h_i}{h_1}$ of an uncompressed and a previously compressed filter cake during pressure stepping experiments (**c**).

We investigated the compression behavior of the filter cake by performing a pressure stepping experiment. The buildup has been executed by applying constant pressure before. For pressure stepping, the pressure is raised three times by 50 mbar and then lowered again three times as illustrated in Fig. 2a. This procedure is repeated twice, such that we distinguish between uncompressed (Fig. 2b) and compressed initial state. To further analyze the cake behavior at elevated pressures, the filter cake thickness change, relative to the initial position 1, $(h_1-h_i)/h_1$, is measured for each pressure step (Fig. 2c). In the uncompressed state, the relaxation does not follow the compression, while in the compressed state, the relaxation follows the compression.

Compared to the results from cake mobility during the buildup, two parallel phenomena occur in this experiment; irreversible rearrangement and elastic compression. The flow-induced rearrangement changes the internal morphology towards an increased order and higher packing density without reversing during relaxation. The elastic compression deforms the soft microgels and reverses during compression. Accordingly, in the initial compression, the filter cake rearrangement dominates step 1 to step 3, while all other steps and the second cycle mainly show elastic deformation. This result underlines the importance of a soft filter cake’s compression history and its decisive influence on its morphology and cake properties.

**Figure 3:**
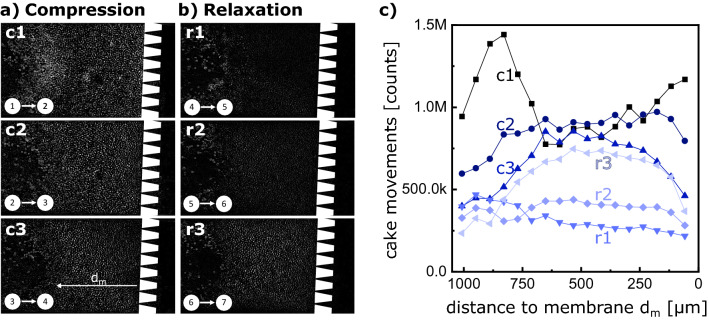
Spacial resolution of the pressure stepping cake mobility. Compression (**a**) and relaxation (**b**) heat maps revealing cake movements during the pressure stepping experiment from Fig. 2 uncompressed. Heat map comparison illustrating spacial resolution of cake movements at different compression and relaxation steps (**c**).

We analyzed the images of the seven compression states in the previously uncompressed cycle. We generated spatial descriptive heat maps, displaying the movements inside the cake (Fig. 3). Therefore, we subtracted the image of a specific compression level from the previous compression level (Fig. 3a,b), such that bright areas in the image highlight the regions with the morphological change, while dark regions are unaffected by the compression and relaxation. Additionally, we plotted these cake movements by accumulating the heat map average pixel counts (Fig. 3c). Accordingly, the Figure shows the brightness in different x-slices in the cake, representing the mobility at that specific position.

The heat map of the first compression c1 is brightest with two peaks at the filter structure (0 $<\,d_m\,<$ 200) and the filter cake surface (750 $<\,d_m\,<$ 1000). At the peak at the membrane structure, the particles rearrange severely by filling up voids. This rearrangement leads to a large change in the total filter cake thickness, as shown in Fig. 2c, step 1-2. These movements are transferred to the following layers, such that the overall brightness is high. The brightness peak at the cake surface is due to a reordering of a very loosely packed layer in front of the cake, which emerged during the initial cake built up at low pressure. In the following compression curves (c2 and c3), the near-membrane peak moves towards the cake center, decreasing the overall brightness. This brightness decrease indicates that rearrangement is reduced and that it mainly occurs distant from the membrane.

During relaxation (r1, r2, and r3) only elastic deformation occurs, such that the heat maps are generally darker. However, the last relaxation step shows a higher movement in all layers compared to the two recent steps. This brightness difference nicely confirms the findings from Fig. 2, where the compression gradient between step 6-7 is more extensive than between step 4-5 and step 5-6.

This analysis confirms the findings from Fig. 2, that the cake movement during compression is more prominent than during relaxation. Additionally, it shows that the rearrangement begins at the membrane surface and evolves towards the center of the cake. The layers close to the membrane experience the highest compression, such that they reach the state of geometrical frustration with exclusive elastic deformation. Further pressure increase enlarges the mechanical agitation and the driving force of particles towards the membrane, such that the layers distant to the membrane also rearrange towards the frustrated state. In relaxation, the frustrated state remains, while the microgels reform elastically towards a sphere.

### CFD-DEM modeling

The microfluidic experiments were accompanied by coupled CFD-DEM simulations investigating the filtration and the compaction of soft particles. Details about the simulation method and conditions are described in the supplementary information. The simulations were performed in a two-step process. First, we filtered soft particles with a constant transmembrane pressure (TMP) of 17 mbar on a particle-impermeable membrane. In the simulations, the particle’s softness is reflected in a low Young’s modulus of 50 kPa, which is adapted from values that can be found in literature for PEG-DA based hydrogels^38–41^. The softness enables the particles to interpenetrate partly.

A hydrodynamic resistance is integrated into the simulation’s CFD part, resulting in a pressure loss in the porous filter cake structure at permeation.

**Figure 4:**
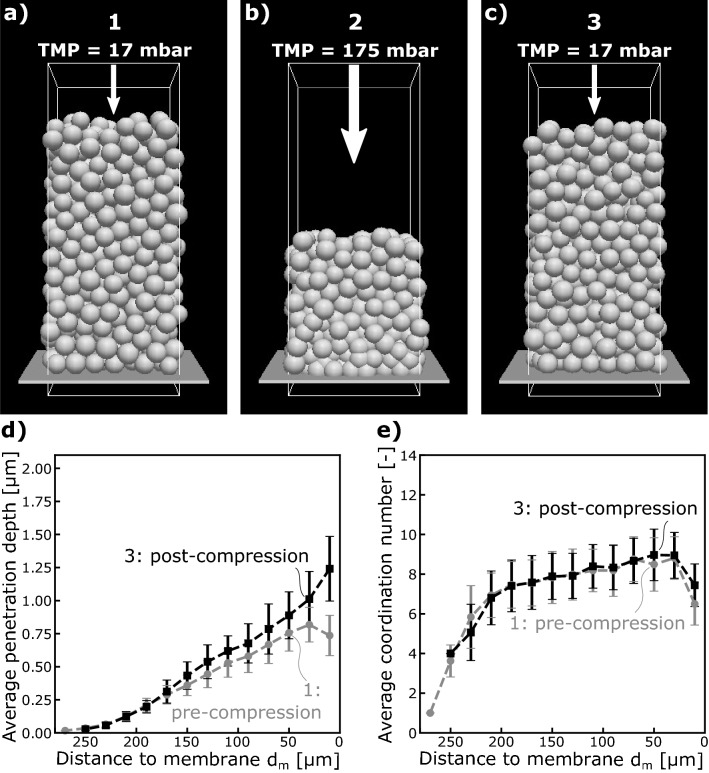
CFD-DEM modeling of cake compression with the visualization of the three conditions (**a**–**c**). The average penetration depth (**d**) and the average coordination number (**e**) of the particles is compared for the pre-compression (grey circles) and the post-compression (black squares) condition.

The particles build up a filter cake with a height of roughly 250 ${\upmu }$m. The resulting filter cake is shown in Fig. 4a. The filter cake exhibits two important characteristics of a soft filter cake: cake compaction accompanied by increased coordination closer to the membrane. Figure 4d (pre-compression) highlights the compaction behavior of the filter cake. The average penetration depth is chosen as a measure for compaction of the filter cake. Since the pressure on the particles raises closer to the membrane, the penetration depth increases. Besides the compaction, the average coordination number increases towards the membrane indicating a more ordered morphology until the coordination number drops off for steric reasons at the membrane boundary.

After filtration, the filter cake is briefly compressed with a higher TMP of 175 mbar. The higher TMP causes the filter cake to compact drastically, as shown in Fig. 4. The cake height roughly reduces to half. After compaction, the filter cake is relaxed again to the TMP of 17 mbar. Back to post-compression steady state, the average penetration depth is increased compared to the pre-compression steady-state, highlighting the filter cake’s irreversible behavior. Further minor changes in the average coordination can be detected. The irreversible compaction shows similarities with the pressure stepping experiments presented in Fig. 2c. In both, the simulation and the experiment, the cake remains more compacted after the compacting step. However, the increased compaction is not as pronounced in the simulation, perhaps due to the simulation domain’s limited size. The avalanche-like, funnel-shaped changes in the filter cake seen in the experiment are restricted to the simulation domain’s limited size. Larger simulation domains would be necessary, but the simulation effort is currently still too high.

## Conclusion and outlook

In this study, we analyzed the movements of soft microgels inside microfluidic filter cakes. First, we fabricated soft spherical PEG microgels by emulsion polymerization. We then filtrated those in microfluidic channels at a porous structure, such that the microgels are fully retained and form a porous filter cake. We localized single-particle movements inside the filter cake during constant pressure cake buildup and classified the movements according to their influence on the overall cake morphology. After rearrangements of single particles or whole particle networks in the filter cake, the deeper layers come to rest, forming a stable set of geometrically frustrated layers. The filter cake stabilizes gradually towards the cake center, starting with the layers close to the membrane.

Additionally, we studied the filter cakes’ compression and relaxation and divided the deformation behavior between a rearranging and an elastic fraction. In the rearranging fraction of the compression, the single microgels fill up voids between other microgels and change their morphological position in the filter cake. In the elastic fraction, the microgels deform while staying in their same morphological position. At the very first compression of a filter cake we find both rearranging and elastic deformation. In contrast, during relaxation and repeated compression, the filter cake only deforms elastically. We furthermore analyze the spatial resolution where the compression and relaxation occurs. We conclude that rearranging compression develops from the near-membrane layers towards the center of the cake, confirming process-known gel layer formation at the membrane boundary. Finally, we confirm our findings of a combined reversible and non-reversible compression by CFD-DEM modeling showing different average penetration depths and coordination numbers before and after a compression step. The presented simulations are a first step to evaluate the microscopic dynamics of soft filter cakes by single particle modeling. In this first phenomenological analysis, we neglect the influence of the surface charge, since the particle’s charge of − 12.7 mV is minor. For future studies, it would be of great interest to refine the model in terms of the influence of surface charge and its interactions on the soft filter cake mobility. Additionally, a more detailed description of the mechanical properties^42,43^ of the particles, including an experimental measurement of the elastic properties^15,44–47^ would be desirable.

This study relates single microgel properties to the overall filter cake behavior and gives novel insights into the events occurring during soft matter filtration inside a filter cake. It underlines the importance of the compression history related to the morphological properties of a soft filter cake and the gel-layer formation process. Additionally, the findings might be used to evaluating soft filter cake models. In the future, further experiments, including flux measurements, might link the here presented cake morphology to the permeation resistance and allow predictions of the impact for real filtration processes.

## Methods

### Fabrication of the microfluidic chip

Two different microfluidic chips were fabricated using soft lithography replica molding. One chip was used for microgel synthesis using emulsion polymerization and one for the filtration experiments. The positive master mold was 3D-constructed using Autodesk Inventor and fabricated by two-photon dip-in direct laser writing following the procedure described by Loelsberg et al.^48,49^. Afterward, PDMS (Sylgard 184, Farnell) was used to replicate the molds, holes for tube connection were punched, and the chip was bonded to a microscope slide with a thickness of 1 mm using oxygen plasma.

### Microgel synthesis

For the microfluidic filtration experiments, we produce PEG-microgels inside a microfluidic chip via emulsion polymerization following the procedure as described elsewhere^50^. In short, the microfluidic cross-junction chip has a height of 50 ${\upmu }$m and a nozzle width of 20 ${\upmu }$m, such that droplets are formed one-by-one at the intersection of two organic and one aqueous phase flow. Due to different manufacturing conditions, the droplet size varies slightly from batch to batch. The aqueous phase contains 10 wt% poly(ethylene glycol) diacrylate (MW 575 g/mol) and 0.5 wt% 2-Hydroxy-4-(2-hydroxyethoxy)-2-methylpropiophenone (Ircagure) as radical photo-initiator. A 50:50 wt mixture of cyclohexane and paraffin oil with 8 wt% SPAN80 is chosen as a continuous organic phase. SPAN80 serves as surfactant and prevents agglomeration of formed droplets. Before synthesis, both solutions are mixed separately for at least 10 min. Polymerization of the emulsion takes place in the outlet tube by exposing the droplet-filled tube to UV-light for at least three minutes. The droplet production rate is adjusted by using a constant pressure microfluidic pump system (Elveflow OB1 MK3). The microgel solution is purified by centrifugation based solvent exchange from cyclohexane to isopropanol and finally to distilled water. The microgels show an average diameter of approx. 27 ${\upmu }$m with a standard deviation of 2.55 ${\upmu }$m and a zeta potential of − 12.7 mV ± 2.95 mV, measured with Zetasizer Ultra (Malvern Panalytical) with a DTS1070 cuvette (Malvern). The slightly negative charge is caused by the remaining acrylate groups in the polymeric network.

### Filtration experiments

The filtration channel is 800 ${\upmu }$m wide and has a height of 150 ${\upmu }$m. 8 filtration blocks with a distance of 3 ${\upmu }$m in between are placed inside the channel to withhold the microgels. Filtration experiments are executed in dead-end mode using a microfluidic constant pressure pump (Elveflow OB1 MK3) and an additional microfluidic pressure sensor Elveflow MPS1 at the chip inlet. The experiments were executed on a light microscope, and the images were analyzed using the imaging software DaVis10 (LaVision GmbH). The channel is at first contacted with water to ensure complete wetting. In a second step, microgels dispersed in distilled water are added to the fluid flow to build a filter cake. The cake buildup experiment (Fig. 1) is conducted by applying constant pressure of 50 mbar to the feed solution. For image analysis a time filter is used to subtract from every image the average of the recent 3 frames for every pixel. This results in highlighted areas illustrating movements in the filter cake. These areas are manually identified and transferred to the graph in Fig. 1c.

The two compression experiments (Fig. 2c) are conducted by first building up a filter cake using 17 mbar feed pressure for 13 min until no flux is visible and no further incoming microgels build up the cake. In this uncompressed state, pressure stepping is performed by increasing and decreasing the pressure in 50 mbar steps. Subsequently, the previously compressed cake undergoes another compression by repeating the compression cycle, which resulted in the “compressed” curve in Fig. 2c The cake thickness is measured at a specific point in the middle of the channel for every pressure state. Inflowing microgels by increased transmembrane pressure and increasing cake thickness are neglected, as each pressure step is executed in a short period of about 5 s. Image analysis for generating the heat-maps (Fig. 3) is conducted by using a time filter and subtracting the intensity of each pixel from the state before and after compression using the “Davis 10” software from LaVision GmbH.

### CFD-DEM simulations

The simulation framework CFDEM$\copyright$ is applied in this study. CFDEM$\copyright$ combines computational fluid dynamics (OpenFOAM$\copyright$) with discrete element method (LIGGGHTS$\copyright$) to describe particle movement inside a fluid flow field^51,52^. In the CFD-DEM approach, individual particle trajectories are determined by a force and momentum balance. The particle motion is coupled with the fluid profile by a momentum exchange enabling two-way coupling between fluid and particle^53,54^. The continuity equation and the volume-averaged Navier-Stokes equation is used to calculate the fluid flow field in the CFD-part. Detailed information about the CFD-DEM coupling algorithm is provided in the supplementary information.

## Supplementary Information

Supplementary Information 1.

Supplementary Information 2.

Supplementary Information 3.

Supplementary Information 4.
