# Supplementary material for: Particle movements provoke avalanche-like compaction in soft colloid filter cakes
Source: Sci Rep. 2021 Jun 18;11:12836. doi: 10.1038/s41598-021-92119-w (PMC8213765; doi:10.1038/s41598-021-92119-w)
Supplement: Supplementary file 1 — Supplementary Information 1. [file 41598_2021_92119_MOESM1_ESM.pdf]

Particle movements provoke avalanche-like compaction in soft colloid filter cakes

Arne Lüken[1,+], Lucas Stüwe[1,+], Johannes Lohaus[1,+], and Matthias Wessling[1,2,*]

[1]RWTH Aachen University, Chemical Process Engineering, Forckenbeckstr. 51, 52074 Aachen, Germany
[2]DWI - Leibniz Institute for Interactive Materials, Forckenbeckstr. 50, 52074 Aachen, Germany
[*]manuscripts.cvt@avt.rwth-aachen.de
[+]these authors contributed equally to this work

Supplementary Information

This supplementary information presents the design files and the CFD-DEM modeling approach used for the simulations of the filter cake compaction and the simulation conditions.

1 Supplementary files

SI1_ChannelGeometry.stl is the CAD-design file of the microfluidic channel that is used for the filtration experiments.

2 CFD-DEM Simulation

The study used the simulation framework CFDEM ©. CFDEM © combines computational fluid dynamics (OpenFOAM ©) with a discrete element method (LIGGGHTS ©) to describe particle motion inside a fluid flow.[1,2] A two way coupling algorithm determines interactions between particle-particle and fluid-particle.

2.1 Discrete Element Method

Discrete element method (DEM) is a Lagrangian simulation method developed by Cundall and Strack.[3] The motion and interactions of particles are solved based on Newton's law of motion. Each particle trajectory is solved by the following force balance:[2]

$$m\ddot{x}_i = F_n + F_t + F_b + F_f \tag{1}$$

where m and $\ddot{x}_i$ are the mass and the acceleration of the particle, respectively. The motion of each particle depends on contact forces (F_n, F_t), body forces (F_b) and forces arising due to the surrounding fluid phase (F_f) .[4]

Hertz contact mechanics is used to determine the tangential and the normal contact forces F_n and F_t:

$$\begin{aligned} F_n &= k_n \delta_n - \gamma_n v_n \\ F_t &= k_t \delta_t - \gamma_t v_t \end{aligned} \tag{2}$$

δn and δt are the normal and tangential overlap of two surfaces. The constants k_n and k_t are the elastic constants and γ_n and γ_t the viscoelastic damping constants. The constants k and γ are material properties depending on the Young modulus E, the Poisson ratio ξ and the coefficient of restitution e.[5] The normal and tangential relative velocity is termed v_n and v_t. The relative tangential velocity includes both the tangential velocity between the spheres and the relative motion due to rotation characterized by the angular velocity.

Besides the Hertzian model, lubrication forces F_lub between the particles are considered in the force balance. If two spheres in a viscous fluid approach each other, the fluid between the spheres has to be squeezed out. The resulting force. When the distance between the surfaces decreases, the pressure gradient necessary to squeeze out the fluid increases. The viscous friction raises through the increased pressure leading to slower relative velocities of the approaching spheres. This lubrication force can be approximated by the following correlation:[6]

$$F = \frac{6\pi\eta v_{rel}}{D}\left(\frac{a_1 a_2}{a_1 + a_2}\right)^2 \tag{3}$$

where η is the fluid viscosity, v_{rel} is the relative velocity between the two surfaces, D is the distance between the two surfaces, a_1 and a_2 are the radii of the two spheres. The lubrication interaction raises to infinity if the separation distance D goes to zero. A cut-off distance $D_0 = 10nm$ is introduced at which the separation distance is assumed to be constant.

A torque balance is applied in addition to the force balance:

$$I\frac{d\omega}{dt} = \sum M \tag{4}$$

where I is moment of inertia, ω is the angular velocity and M are the contact torques. This study applied the constant direct torque model to account for rolling friction,[7] which is determined as follows:

$$M_{rf} = C_{rf} k_n \delta n r \frac{\omega_{rel,shear}}{|\omega_{rel,shear}|} \tag{5}$$

where C_{rf} defines the coefficient of rolling friction and $\omega_{rel,shear}$ is the projection of the relative angular velocity into the shear plane.

2.1.1 Unresolved CFD-DEM approach

The volume averaged CFD-DEM approach is used for modeling the motion of the particle-laden incompressible fluid phase. The volume-averaged Navier-Stokes equations, consisting of the continuity equation

$$\frac{\partial(\alpha_\mathrm{f})}{\partial t} + \nabla\cdot(\alpha_\mathrm{f} u) = 0 \tag{6}$$

and the momentum equation are applied[2]

$$\frac{\partial(\alpha_\mathrm{f} u)}{\partial t} + \nabla\cdot(\alpha_\mathrm{f} uu) = -\alpha_\mathrm{f}\frac{\nabla p}{\rho_\mathrm{f}} - R_\mathrm{pf} + \nabla\cdot\tau, \tag{7}$$

where α_f denotes the void fraction of the fluid, u the velocity of the fluid, ρ_f the fluid density, p the pressure, R_pf the momentum exchange between particle and fluid, and $\tau = \nu(\nabla u)^T$ the stress tensor with the kinematic viscosity ν. A porous layer is integrated into the CFD part of the simulations representing the pressure drop caused by pore structure in the experiments. The hydrodynamic resistance of the porous layer is added to the Navier-Stokes equation as follows:

$$\frac{\partial \varepsilon u_f}{\partial t} + \nabla\cdot(\varepsilon u_f u_f) = -\varepsilon\nabla\frac{p}{\rho_f} + \nabla\cdot\tau - R_{pf} + S_m \tag{8}$$

$$S_m = -\nu k_M u_f \tag{9}$$

The resistance k_M is chosen to reflect the hydrodynamic resistance of the microfluidic experiments.

The momentum exchange R_pf is calculated with the following equation:[2]

$$R_\mathrm{pf} = K_\mathrm{pf}(u - \langle v\rangle), \tag{10}$$

with

$$K_\mathrm{pf} = -\frac{|\sum_i F_{\mathrm{D}_i}|}{\rho_\mathrm{f} V_\mathrm{cell}|u-\langle v\rangle|}. \tag{11}$$

The momentum exchange is calculated for each CFD cell with the volume V_cell, based on the difference between the fluid velocity u and the volume averaged particle velocity $\langle v\rangle$.

The drag correlation based on the work of Gidaspow is chosen:[8]

$$F_d = \beta_{pf}/\rho_f(u_f - u_p) \tag{12}$$

The drag pre-factor β_{pf} decreases with the void-fraction ε of the fluid phase:

$$\beta_{pf} = \begin{cases} \frac{3}{4}C_d\frac{\rho_f(1-\varepsilon)|u_f-u_p|}{d_p}\varepsilon^{-2.65}, & \varepsilon > 0.8 \\ 150\frac{(1-\varepsilon)^2\nu_f}{\varepsilon d_p^2} + 1.75\frac{(1-\varepsilon)|u_f-u_p|\rho_f}{d_p}, & \varepsilon \leq 0.8 \end{cases} \tag{13}$$

The divided approach is used to calculate the fluid void fraction in a CFD cell.[9] The divided approach splits the particle volume in 29 of equally sized elements. The center point of each element is calculated and the solid fraction is spread over the mesh elements.

Additional smoothing of the void fraction is applied to improve stability of the particle-liquid coupling.[10] The isotropic diffusive smoothing can efficiently be applied to smooth the void fraction field and the momentum exchange term. The following equation is solved for both variables which are represented ξ:

$$\frac{d\xi}{dt} = \nabla^2\left(\frac{\lambda^2}{\Delta t_{CFD}\xi}\right) \tag{14}$$

The isotropic smoothing method is controlled with the smoothing length λ, which is chosen to be the mean particle's diameter of 20 µm.

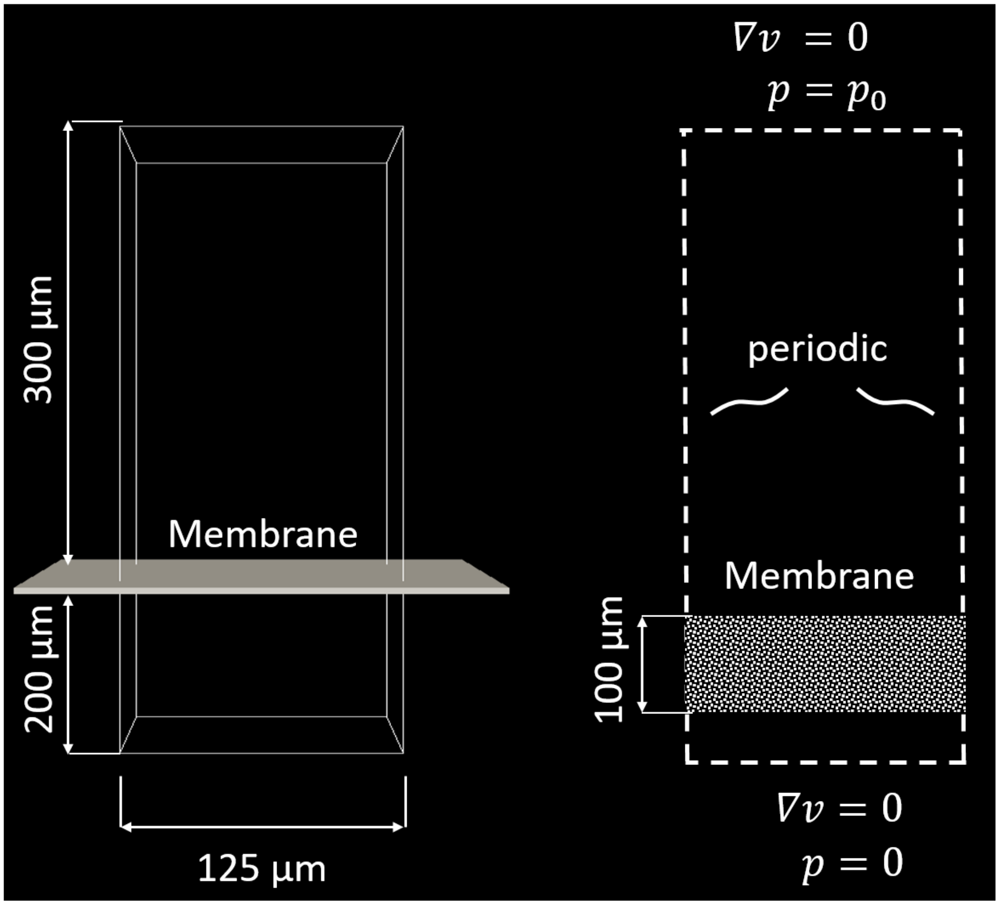

Figure 1. Simulation domain and boundary conditions

3 Simulation domain and conditions

The simulations were performed in a cuboid including a porous structure, which is shown in Figure 1. The mesh is chosen to be a structured mesh of 25x25x25 µm ensuring the particle size to be smaller than the mesh size. Microgels were filtered on a particle-impermeable layer. The size of the microgels varies over a Gaussian distribution of 18 to 22 micrometers with an average value of 20 micrometer. The particles were filtered with a constant transmembrane pressure (TMP) of 17 mbar until the filter cake reached a height of roughly 250 µm. After filtration, the filter cake is shortly exposed to a TMP of 175 mbar leading to compression of the filter cake. After compression, the cake is relaxed by reducing the TMP again to 17 mbar until steady state is reached. The simulations lasted up to two weeks on eight cores on an AMD Ryzen Threadripper 2990WX processor

3.1 Data analysis

The simulation results were analyzed with a python script to gain more information about the cake compression and the cake morphology.

To calculate the filter cake compaction, the filter cake was subdivided into regions of 20 µm in the z-direction. The distance between contacting spheres was calculated under consideration of the periodic boundaries. The resulting contact distances were averaged for each segment, and the standard deviation was calculated. Hence, the degree of compaction can be measured depending on the distance to the membrane.

The coordination number of the particles was calculated to analyze the cake packing. The coordination number describes the number of contacts of each particle. Equal sized spheres in crystalline regions posses in close-packing a coordination number of 12. Again, the particle's location was subdivided into regions of 20 µm in the z-direction. Thereby, the degree of coordination is measured depending on the distance to the membrane.

Parameter	Symbol	Value
Particle diameter	σ	$18-22$ µm
Young modulus	E	50 kPa
Feed particle concentration	c	30 $vol\%$
Poisson ratio	$\tilde{\nu}$	0.4
Coefficient of restitution	e	0.6
Membrane resistance	k_M	40 μm^{-2}
Lubrication separation distance	D_{lub}	10 nm
DEM-time step	Δt_{DEM}	4×10^{-9} s
CFD-time step	Δt_{CFD}	2×10^{-8} s

Table 1. Parameters applied in the simulations

References

1. Goniva, C., Kloss, C., Hager, A. & Pirker, S. An open source cfd-dem perspective. In *Proceedings of OpenFOAM Workshop, Göteborg*, 22–24 (2010).
2. Kloss, C., Goniva, C., Hager, A., Amberger, S. & Pirker, S. Models, algorithms and validation for opensource dem and cfd–dem. *Progress in Computational Fluid Dynamics, an International Journal* **12**, 140–152 (2012).
3. Cundall, P. & Strack, O. A discrete numerical model for granular assemblies. *geotechnique* **29**, 47–65 (1979).
4. Zhu, H., Zhou, Z., Yang, R. & Yu, A. Discrete particle simulation of particulate systems: theoretical developments. *Chemical Engineering Science* **62**, 3378–3396 (2007).
5. Di Maio, F. & Di Renzo, A. Modelling particle contacts in distinct element simulations: Linear and non-linear approach. *Chemical Engineering Research and Design* **83**, 1287–1297 (2005).
6. Lisicki, M. & Nägele, G. Colloidal hydrodynamics and interfacial effects. In *Soft Matter at Aqueous Interfaces*, 313–386 (Springer, 2016).
7. Ai, J., Chen, J.-F., Rotter, J. & Ooi, J. Assessment of Rolling Resistance Models in Discrete Element Simulations. *Powder Technology* (2011).
8. Gidaspow, D., Bezburuah, R. & Ding, J. Hydrodynamics of circulating fluidized beds: kinetic theory approach. Tech. Rep., Illinois Inst. of Tech., Chicago, IL (United States). Dept. of Chemical ... (1991).
9. Norouzi, H., Zarghami, R., Sotudeh-Gharebagh, R. & Mostoufi, N. *Coupled CFD-DEM Modeling: Formulation, Implementation and Application to Multiphase Flows* (John Wiley & Sons, 2016).
10. Blais, B., Lassaigne, M., Goniva, C., Fradette, L. & Bertrand, F. Development of an unresolved cfd-dem model for the flow of viscous suspensions and its application to solid-liquid mixing. *Journal of Computational Physics* **318**, 201–221 (2016).

Additional information

The authors declare no competing interests.
